# Comparison of Risk of Preterm Labor between Vaginal Progesterone and17-Alpha-Hydroxy-Progesterone Caproate in Women with Threatened Abortion: A Randomized Clinical Trial 

**DOI:** 10.22074/ijfs.2016.4905

**Published:** 2016-06-01

**Authors:** Abootaleb Beigi, Arezoo Esmailzadeh, Reyhane Pirjani

**Affiliations:** Department of Obstetrics and Gynecology, Arash Maternity Hospital, Tehran University of Medical Sciences, Tehran, Iran

**Keywords:** Progesterone, 17-Alpha-Hydroxyprogesterone Caproate, Premature Labor, Threatened Abortion

## Abstract

**Background:**

Threatened miscarriage is a common complication in pregnancy that leads to adverse pregnancy outcomes such as preterm labor. This study aimed to compare the vaginal progesterone (Cyclogest) versus 17-alpha-hydroxyprogesterone caproate (Proluton) on preventing preterm labor in pregnant women with threatened abortion at less than 34 weeks’ gestational age.

**Materials and Methods:**

This balanced randomized, double-blind, single-center controlled clinical trial included 190 women with threatened abortion. They were then randomly allocated into Cyclogest (n=95) and 17-alpha-hydroxyprogesterone caproate (Proluton, n=95) groups. Interested outcome was preterm labor less than 34 weeks. The Pearson chi-square and Student’s t test were used to compare two groups. The data were analyzed by Stata software version 13.

**Results:**

The risks of preterm labor less than 34 weeks in Proluton and Cyclogest groups were 8.6 and 6.52%, respectively. There was no significant difference for risk of preterm labor less than 34 weeks [relative ratio (RR): 1.31, 95% confidence interval (CI): 0.47- 3.66, P=0.59] between two groups.

**Conclusion:**

Risk of preterm labor in the vaginal progesterone group and 17-alpha-hydroxyprogesterone caproate group in pregnant women with threatened abortion is the same (Registration Number: IRCT2014123120504N1).

## Introduction

Preterm labor is defined as babies born alive at less than 37 weeks’ gestational age (259 days), (1,3) which is divided into three groups, including extremely preterm (less than 28), very preterm (28 to less than 32 weeks) and moderate to late preterm (32 to less than 37 weeks) (1). Based on the study by Blencowe et al. (4), 14.9 million babies are born worldwide before 37 weeks’ gestational age, indicating about 11.1% of all live births, which is more than 1 in 10 babies. Preterm labor rates across 184 countries in the world range from 5 to 18 % (5). Also, preterm labor rates in Iran were estimated from 5.1-8.4% (6,7). 

The preterm labor is the most important cause of neonatal mortality and morbidity, estimated 27% of neonatal deaths, and several studies have revealed mortality and morbidity rates are inversely associated with gestational age at delivery time (3,8,12). Several risk factors are related to preterm labor such as shortened cervical length, infection, previous preterm labor, socioeconomic status, nutritional status, threatened abortion, etc. (13,15). Threatened abortion refers to vaginal bleeding before 20 weeks’ gestational age and occurs in 20% of pregnancy (a fifth of cases) (16,18). 

Progesterone has been shown to be an important factor for maintaining uterine quiescence in the latter half of pregnancy, probably by restricting the production of prostaglandins and inhibiting the expression of contraction-associated protein genes within the myometrium, including oxytocin and prostaglandin receptors, ion channels and gap junctions (19,20). The weeks before labor, levels of progesterone in the maternal circulation remain unchanged, although the onset of labor is directly associated with a functional withdrawal of progesterone activity (20,22). In both basal and pro-inflammatory conditions, progesterone stops apoptosis in fetal membrane explants (23). This may help to prevent preterm premature rupture of membranes, which is a frequent cause of preterm birth. However, application of progesterone has the US Food and Drug Administration (FDA) approval in preterm labor. In liver, progesterone is metabolized through reduction to pregnanetriol, pregnanediol, and pregnanolone, and it was then excreted through the urine. It is noted that function of progesterone receptors are important for development of pregnancy, while the serum level of progesterone less than 45 nmol/l is considered as useful tool to predict nonviable pregnancy (24). 

Moreover, a systematic review and meta-analysis of randomized controlled trials showed that there is a significant reduction in risk of preterm delivery when using progestational agents (25). Additionally, several studies revealed that administration of progesterone in women with threatened abortion increases the fetus survival rate (26,28). 

Threatened miscarriage is known as a risk factor for preterm labor. In a number of studies such as Hassan et al. (29) and Meis et al. (30) in which they applied weekly injections of 250 mg of 17P starting from weeks 16 to 36 (delivery), and Foneseca et al. (31) in which they applied vaginal progesterone (200 mg/each night) from weeks 24 to 34, a long-term progesterone therapy is considered as a preventive therapy for preterm labor. In this study, spontaneous delivery before 34 weeks of gestation was less common in the progesterone group than in the placebo group [19.2 vs. 34.4%, relative risk (RR): 0.56], indicating that treatment with progesterone prevents spontane- ous preterm birth in women with a short cervix. Also, in a multicenter randomized controlled trial conducted by Grobman et al. (32), they revealed that there is no significant difference regarding the frequency of preterm birth between the 17-alpha-hydroxyprogesterone caproate (225.1%) and placebo groups (24.2%). 

Progesterone is usually given as injection and vaginal suppository forms that are based on a patient’s choice. Therefore, this study aimed to compare the vaginal progesterone (Cyclogest) versus injection of 17-alpha-hydroxyprogesterone caproate (Proluton) in preventing preterm labor in pregnant women with threatened abortion at less than 34 weeks’ gestational age. 

## Materials and Methods

### Trial design

This study was a balanced randomized, doubleblind, single-center controlled clinical trial. Eligible patients were recruited from the women with threatened abortion referred to the Department of Gynecology and Obstetrics, Arash Hospital, Tehran, Iran, from January 2014 to March 2015. The study was approved by Institutional Review Board for Human Research of Tehran University of Medical Sciences (Ethical Committee code: 93/d/130/556). Patients were informed about the aim of the study as well as benefits and harms of treatments before being randomly allocated into two groups. An informed consent was obtained from all patients. The trial was registered on the Iranian website for registration. 

### Eligibility criteria

The inclusion criteria were as followings: i. Genital abnormalities, ii. Confirmed singleton pregnancy, iii. Between 6 and 20 weeks’ gestational age at enrollment time, and iv. Threatened abortion ( vaginal bleeding or any bloody vaginal discharge during the first half of pregnancy. Exclusion criteria were as followings: i. Uncontrolled medical diseases (hypertension, diabetes, cardiovascular, renal or hepatic disease), ii. History of drugs or alcohol abuse, iii. Lack of pregnancy sac at week 5, iv. Lack of yolk sac at weeks 5.5-6, v. Lack of fetus at weeks 6-6.5, vi. Lack of fetal heart rate at 16-24 weeks of gestational age, vii. Cervical length of less than 25 mm that was measured by transvaginal ultrasound at 16-24 weeks, viii. Fetal abnormalities, ix. History of previous preterm delivery, and x. Placenta previa. Transvaginal ultrasound was done by an expert radiologist for all patients in the clinic (free of charge). 

### Sample size

Based on an expected occurrence of the primary endpoint (preterm labor) of 6% in the Proluton group and 20% in Cyclogest group, we estimated that a minimum sample size of 91 patients in each treatment group is necessary to give 80% power to detect a significant difference between two groups (with a two-sided type I error of 5%). Our hypothesis was that Proluton would decrease the preterm labor rate in patients compared to Cyclogest. Therefore, 190 patients were randomly assigned into two groups [Cyclogest (n=95) and Proluton; n=95)], and five patients were then excluded from the analyses because they failed to meet inclusion criteria. Finally, 185 patients were included in the analysis [Cyclogest group (n=93) and Proluton group (n=92)]. 

### Randomization

Patients (n=190) were randomly assigned into two groups, Cyclogest (n=95) and 17-alphahydroxyprogesterone caproate (Proluton, n=95) groups, using a computer-generated program that was provided by a statistician, who was independent of the clinical study team. To avoid imbalance of patients in two groups during study, randomization was performed in blocks of six (three received either Cyclogest or Proluton). 

To ensure allocation concealment, an independent subject prepared the randomization list and the sequence was protected in a sealed envelope. Whenever a patient was found qualified, the numbered envelope was opened to determine the intervention technique. The outcome assessors, caregiver and data analysts were blinded to the assigned treatment throughout the study

### Interventions

 The Proluton group (n=92) underwent a weekly administration of 17-α-hydroxyprogesterone caproate, 250 mg/intramuscular injection/weekly. The Cyclogest group (n=93) was treated daily with vaginal progesterone suppository at a dosage of 400 mg (in the analysis, vaginal progesterone was considered as baseline group). They were applied every night to the end of week 34 of gestation. Patients were trained to use the suppositories. The medications were supplied by the pharmacy of Arash Hospital and administrated by the ward nurse who was blinded to the study. The patients were registered and followed up in the hospital using an electronic record of the prenatal clinic. Data gathering was done by two educated residents. 

### Outcome measures

At study entry, a trained physician performed standardized interviews that included information about demographic and clinical data. In addition, maternal weight and height were assessed and a routine laboratory work-up was carried out. The primary outcome measure was the preterm labor. Preterm refers to a birth that occurs at less than 34 weeks of gestation. Gestational age was calculated on the basis of the last menstrual period and the results of ultrasound scan, indicating the crownrump length and the gestational sac dimension. Postoperative side effects related to two medications were recorded. 

### Statistical analysis

Categorical and continuous variables were summarized as proportions and mean ± SD, respectively. The Pearson chi-square and Student’s t test were used to compare variables at baseline between two groups based on type of variables. 

Results are presented as RR, 95% confidence interval (CI). The association was considered significant when the P value was <0.05 or when the 95% CI for RR did not include 1.0 (equivalent to P<0.05). Data analysis was undertaken using the SPSS (SPSS Inc., USA) version 13.0. 

### Results

A total of 223 women were interviewed, of which thirty-three women did not meet the inclusion criteria and 190 were eligible to participate in this study, so they were randomly assigned into Proluton (n=95) and Cyclogest (n=95) groups. Five women were then excluded from the analyses because they failed to complete the study, so 185 participants completed the study. The statistical analysis was also performed according to an intention to treat approach. 

The distribution of demographic data and pregnancy history variables are reported in Table 1, indicating that no significant differences were found between the two groups of patients on the basis of age, body mass index (BMI), gestational age at enrollment, cervical length in the third trimester, history of abortion, history of ectopic pregnancy and parity. 

**Table 1:** Baseline demographics and clinical characteristics of participants randomly allocated

Variables	Cyclogest group n=92	Prolutongroup n=93	P value
Age (Y)^*^	28.37 (5.21)	28.56 (5.31)	0.8
BMI^*^	25.59 (4.83)	24.68 (4.05)	0.17
Marital duration^*^	5.98 (4.98)	4.85 (0.73)	0.07
Gestational age at enrollment^*^	11.15 (5.35)	10.96 (6.02)	0.82
Cervical length in the second trimester^*^	28.03 (5.34)	28.3 (6.73)	0.79
Parity^*^	0.15 (0.44)	0.2 (0.71)	0.5
History of ectopic pregnancy #			
Yes	4 (4.34)	2 (2.15)	
No	88 (95.66)	91 (97.85)	0.39
History of abortion #
Yes	24 (26.08)	20 (21.5)	0.46
No	68 (73.92)	73 (78.5)	

BMI; Body mass index, ^*^; Data are presented as mean (SD) for continuous variables, and ^#^; Number (percent) for categorical variables.

As shown in Table 2, the number of preterm labor less than 28 completed weeks in women treated with Proluton was 6 out of 93 (6.45%), whereas in the Cyclogest group, there were 4 out of 92 (4.35%). The number of preterm labor less than 34 completed weeks in Proluton group was 8 out of 93 (8.60%), whereas there were 6 out of 92 (6.52%) in the Cyclogest group, and also preterm labor less than 37 completed weeks was observed in 16.30% of Cyclogest group and 15.05% of Proluton group. There were no sig- nificant differences regarding risk of preterm labor less than 28 completed weeks (RR: 1.48, 95% CI: 0.43-5.10, P=0.53), preterm labor less than 34 completed weeks (RR: 1.31, 95% CI: 0.47-3.66, P=0.59), and preterm labor less than 37 completed weeks (RR: 0.92, 95% CI: 0.47- 1.80, P=0.81) between two groups.

**Table 2:** Relative risk (RR) and 95% confidence interval (CI) of pre- term labor in two groups


Preterm labor	Risk of preterm labor*		RR (95% CI)	P value
	Cyclogest group	Proluton group		

Less than 28 weeks	0.043 (4)	0.064 (6)	1.48 (0.43-5.08)	0.52
Less than 34 weeks	0.065 (6)	0.086 (8)	1.31 (0.47-3.65)	0.59
Less than 37 weeks	0.163 (15)	0.150 (14)	0.92 (0.47-1.80)	0.81


*; Data are presented as percent (number).

## Discussion

The present study compared the risk of preterm labor between vaginal progesterone (Cyclogest) and 17-alphahydroxy-progesterone caproate (Pro- luton). The results of this study indicated that there is no statistically and clinically significant differ- ence in risk of preterm birth (less than 34 weeks) between Cyclogest and Proluton groups. Also, there was no significant difference in risk of pre- term labor less than 28 completed weeks and pre- term labor less than 37 completed weeks between two groups.

Threatened abortion occurred in one of five preg- nancies and 10% of women with threatened abortion will experience abortion (16). Progesterone decreas- es preterm labor and abortion rates via suppressing luteinize hormone in luteal phase and improving im- plantation and regulation of immune responses, while progesterone decreases the prostaglandins synthesis and inhibits inappropriate contractions of uterus.

In this study, the risks of preterm labor less than 34 weeks in women with and without history of abortion were 0.068 and 0.078, respectively, and there is no significant difference between two groups. Tita and Rouse (33) in a review study showed that the 17-alpha-hydroxyprogesterone effectively reduces the incidence of recurrent preterm labor in women with a history of spontaneous preterm labor. Also in a study by Meis et al. (30), they declared that weekly injections of 17-alpha-hydroxyprogesterone led to considerable reduction in the recurrent preterm delivery rate among high risk women for preterm delivery. 

In the previous study, women were enrolled at 19 clinical centers at 16 to 20 weeks of gestation and randomly assigned to receive weekly injections of 250 mg of 17P that injections were continued until delivery or to 36 weeks of gestation (a long time progesterone therapy) (32). Also in the study by Fonseca et al. (31), spontaneous delivery before 34 weeks of gestation was less frequent in the progesterone group receiving vaginal progesterone (200 mg each night) from 24 to 34 weeks of gestation. However, the results of previous studies are inconsistent with each other. Some studies stated that 17-alpha-hydroxyprogesterone had no additional advantage for prevention of preterm labor in women with prior spontaneous preterm labor and did not improve neonatal and maternal outcomes (34). Negligible adverse effects are related to the route of administration, and include injection site reactions and vaginal discharge. The only important side effect that reported in some studies is a threefold increase in risk of developing gestational diabetes (35), but this finding was not confirmed in large data study (36). The main concern about synthetic progesterone is binding to androgen receptors, but 17-alpha-hydroxyprogesterone as a natural progesterone metabolite is produced by the corpus luteum and placenta with minimal to no androgenic activity. One concern is a probable increase in risk of hypospadias in male offspring exposed to exogenous progestins (37). The benefit of vaginal progesterone is its high uterine bioavailability since uterine exposure occurs earlier than pass through the liver. 

In a systematic review and meta-analysis by Mackenzie et al. (25), they revealed that use of progestational agents, initiated in the second trimester of pregnancy, for women at risk of preterm labor possibly decreases the rate of preterm labor, but the effect of progestational agents on neonatal outcome is doubtful. In contrast, a study by Chawanpaiboon et al. (38) conducted on 150 pregnant women with threatened preterm labor between 28 and 35 weeks of gestation revealed progesterone is not the most effective agent as compared to other agents (Nifedipine) and also bed rest. 

In this study, in group treated with Cyclogest, eclampsia was observed only in two cases, IUGR in two cases, and membrane rupture in one case, whereas in the group treated with Proluton, no adverse effects were reported, indicating that complications arising as side effects are not attributable to progesterone agents. The observed effects in this group can be caused by other factors. 

As regards, there was no difference between two groups in term of preterm labor risk reduction, whereas vaginal progesterone (Cyclogest) is safe for mother and fetus, not painful, cost effective and more compliance, suggesting that Cyclogest is highly recommended as compared with Proluton. 

Due to rarity of preterm labor, further studies with more sample size or multicenter design are, therefore, suggested in order to have an appropriate statistical power. 

One of the limitations of this study was the lack of a control group which was not possible for ethical reasons. Also some other unmeasured confounder variables like stress, anxiety, family history of abortion and preterm labor may affect the results. In this study, the interested outcome was an objective outcome, while the lack of patients blinding to the treatment results in less complication for subjective outcomes. 

## Conclusion

Based on the findings, there was no significant difference in the risk of preterm labor between the vaginal progesterone (Cyclogest) and 17-alphahydroxy progesterone caproate (Proluton). 
